# Protective Effect of Buyang Huanwu Decoction on Cerebral Ischemia Reperfusion Injury by Alleviating Autophagy in the Ischemic Penumbra

**DOI:** 10.1155/2021/9937264

**Published:** 2021-12-07

**Authors:** Yanmeng Zhao, Xiujuan Ma, Wentao Yu, Ziwei Zhang, Wenliang Wang, Xiaohong Zhou, Weijuan Gao

**Affiliations:** ^1^Hebei Key Laboratory of Chinese Medicine Research on Cardio-Cerebrovascular Disease, Hebei University of Chinese Medicine, Shijiazhuang 050200, China; ^2^Affiliated Hospital of Chengde Medical University, Chengde Medical University, Chengde 067000, China

## Abstract

**Objectives:**

To evaluate the protective effect of Buyang Huanwu Decoction (BHD) against cerebral ischemia reperfusion and investigate whether autophagy is involved in its mechanism of action.

**Methods:**

Adult male Sprague Dawley rats were randomly divided into three groups: the sham, cerebral ischemia reperfusion (I/R), and I/R + BHD groups. A rat model of cerebral I/R injury was established via middle cerebral artery occlusion (MCAO) for 2 h, followed by 1, 3, and 7 d of reperfusion. Neurological scores and regional cerebral blood flow were assessed to determine whether the model was successfully established. Brain infarct volume was determined by 2,3,5-triphenyl tetrazolium chloride (TTC) staining. The apoptosis rate was detected using TdT-mediated dUTP Nick-End Labeling (TUNEL) staining, and neuronal damage was evaluated by Nissl staining. The Beclin-1 and LC3 protein levels in the ischemic core, penumbra, and contralateral area were analysed by Western blotting. The occurrence of autophagy in the penumbra was observed by transmission electron microscopy (TEM).

**Results:**

BHD treatment alleviated the cerebral infarct volume, neuronal apoptosis rate, and neuronal damage 3 and 7 d after cerebral I/R injury. Furthermore, 3 d after reperfusion, we observed that the Beclin-1 levels were significantly decreased in the core in the I/R group, whereas transformation of LC3 I to LC3 II exhibited no obvious differences between the sham and I/R groups. In the penumbra, the Beclin-1 levels and transformation of LC3 I to LC3 II in the I/R group were significantly increased compared with that in the sham group. However, no significant difference in the contralateral area was noted between the two groups. BHD significantly inhibited the expression of Beclin-1 and the transformation of LC3 I to LC3 II in the penumbra after cerebral I/R injury but yielded no significant changes in the core and contralateral area.

**Conclusions:**

BHD exerts a neuroprotective effect by inhibiting autophagy in neurons in the penumbra after cerebral I/R injury.

## 1. Introduction

Stroke is one of the leading causes of death and disability worldwide, and 60–80% of stroke cases are ischemic stroke [1, 2]. Cerebral I/R injury often occurs during the treatment of stroke, aggravating brain injury; however, no effective clinical treatment is available for cerebral I/R injury [1, 3, 4]. Accumulating evidence shows that autophagy is activated in cerebral I/R injury, and regulation of the autophagy process may represent a potential therapeutic strategy in the treatment of cerebral I/R injury [5–7].

Autophagy is a process of cell “self-eating” whereby cell constituents are delivered to the lysosome for digestion and recycling [2]. Autophagy normally functions to promote cell survival, but excessive autophagy can lead to cell death called “autophagic cellular death” [7]. The proper execution of autophagic programmes requires Atg proteins [7]. As Atg proteins, Beclin-1 and LC3 play an important role in the autophagy regulation process and are also key indicators for detecting autophagy levels. Beclin-1 promotes autophagosomal membrane nucleation. LC3 II is tightly correlated with the number of autophagosomes and is considered the most reliable marker of active autophagosomes and autolysosomes [8].

It has been demonstrated that autophagy occurs in the MCAO/reperfusion (MCAO/R) model of rats and mice [9, 10]. However, whether autophagy is protective or pathological in MCAO/R conditions remains controversial. Some studies have demonstrated that the induction of autophagy is a potential therapeutic strategy in the treatment of cerebral I/R injury [11, 12]. However, other studies have noted that cerebral I/R injury induces excessive autophagy damage to the brain [13, 14]. The degree of autophagy activation may be related to the species of experimental animals and the time of ischemia and reperfusion. Brain lesions associated with cerebral I/R injury can be conceptually divided into the core and penumbra [2]. However, the occurrence of autophagy in different brain areas has rarely been reported.

Traditional Chinese medicines (TCMs) have certain advantages in the treatment of cerebral I/R injury given their multitarget effects and limited side effects. Buyang Huanwu Decoction (BHD) is a well-known classical prescription in TCM that is used for the treatment of stroke [15]. The prescription of BHD was first recorded in the “Yi Lin Gai Cuo (Correction on Errors in Medical Classics)” by Wang Qingren in 1830 [16]. BHD replenishes Qi, activates blood circulation, and dredges collaterals, and it has a significant therapeutic effect on stroke [17]. Basic medical studies have shown that BHD has neuroprotective effects on animal models of cerebral I/R injury through anti-inflammatory, antiapoptosis, and proangiogenesis effects and other mechanisms [18–21]. However, the regulatory effect of BHD on autophagy and in different areas of the ischemic brain is rarely studied.

In this study, we investigated the protective effects of BHD against cerebral I/R injury at different time points and further explored the underlying treatment mechanism, which involves the regulation of autophagy in different injured areas of the brain.

## 2. Materials and Methods

### 2.1. Animals

Male Sprague Dawley (SD) rats (250–280 g) were purchased from Beijing Vital River Laboratory Animal Technology Co. Ltd. (China, license number: SCXK (Jing) 2016-0006). Food and water were provided ad libitum throughout the experiment. All animal experiments were performed strictly in accordance with the National Institutes of Health Guide concerning the care and use of laboratory animals and were approved by the Animal Ethics Committee of Hebei University of Chinese Medicine (approval number: DWLL2018041).

### 2.2. Preparation and Quality Control of BHD

BHD was purchased from the Beijing Tongrentang drug store and was composed of the following dried herbal components: 120 g of Radix Astragali, 6 g of Radix Angelicae Sinensis, 5 g of Radix Paeoniae Rubra, 3 g of Ligusticum Wallichii, 3 g of Flos Carthami, 3 g of Semen Persicae, and 3 g of pheretima. A mixture of the herbal components of BHD was soaked in distilled water for 30 min to soften the raw materials and facilitate extraction of water-soluble constituents. Then, the components were boiled with high heat to boil and gently for 30 min, twice. The suspension obtained by two decoctions was mixed evenly, concentrated to 100 ml, and stored at 4°C for subsequent experiments.

The quality control assessment of BHD by high-pressure liquid chromatography-mass spectrometry (HPLC-MS) was performed on a Shimadzu LC-30A HPLC system with AB SCIEX QTRAP 4500 mass spectrometry. The analytes were separated on a Shim-pack GIST C18 column (2.1 × 100 mm, 2 *μ*m) at 40°C. The mobile phase was composed of 0.1% formic acid in water (solvent A) and acetonitrile (solvent B). The flow rate was 0.4 ml/min and the injection volume was 0.5 *μ*l. For quantitation, a mixed stock solution was prepared by dissolving astragaloside IV (11.7 *μ*g/ml), paeoniflorin (51.8 *μ*g/ml), calycosin-7-glucoside (22.3 *μ*g/ml), and ferulic acid (5.325 *μ*g/ml) in methanol. BHD (1 ml) solution was diluted to 10 ml with methanol and filtered through a 0.45 mm membrane filter. The contents of the four ingredients were calculated via a one-point external standard method, and the assays were repeated twice.

### 2.3. MCAO Model Establishment and Drug Treatment

The rats were anesthetized with 1% pentobarbital sodium by intraperitoneal administration (50 mg/kg) and then placed on an operating table at a constant temperature. An incision was made on the centre left of the neck. The left common carotid artery (CCA) and external carotid artery (ECA) were exposed, isolated, and ligated, and the ECA was severed. The middle cerebral artery (MCA) was occluded by inserting a monofilament with a rounded tip into the internal carotid artery (ICA) until it reached the origin of the MCA. After 2 h of ischemia, reperfusion was implemented by withdrawal of the monofilament. A perfusion speckle imager (PeriCam PSI Z, Perimed AB, Sweden) was used to monitor the regional cerebral blood flow before ischemia, 5 min after ischemia, and 5 min after reperfusion. Rats with blood flow that was reduced to 35% of the baseline and restored to 80% of baseline after reperfusion were included in this study. Neurological scores were evaluated 2 h after reperfusion by a blinded observer using the Zea Longa 5-point scoring system: 0 points, normal performance with no neurological deficits; 1 point, contralateral forepaws not able to fully extend; 2 points, circling to the opposite side when walking; 3 points, falling to the opposite side when walking; and 4 points, no spontaneous walking and loss of consciousness. Of these, rats with 1 to 3 points were included as experimental subjects. Rats with 0 or 4 points and dead rats were removed from the analysis.

**Figure 1:**
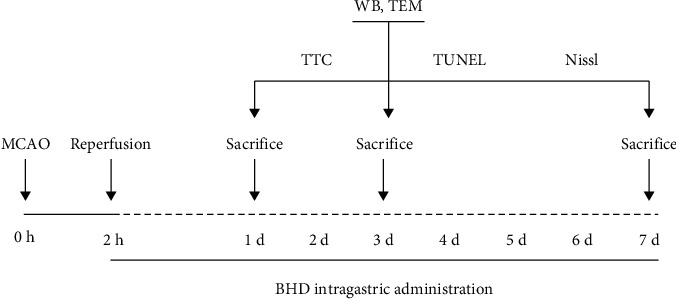
Time schedule of experimental procedures.

Rats were randomly divided into 3 groups: sham group, I/R group, and I/R + BHD group. The rats underwent MCAO operation in the I/R group and I/R + BHD group, whereas the sham rats were subjected to the same operation but without MCAO. In the I/R + BHD group, rats were administrated 14.3 g/kg BHD by gavage 2 h after reperfusion and every 24 h later. The decoction was administrated continuously for 1, 3, and 7 d (Figure 1).

### 2.4. Measurement of Infarct Volume

Three rats from each group were anesthetized with 1% pentobarbital sodium (50 mg/kg, intraperitoneally) at 1, 3, and 7 d after reperfusion, and the whole brains were quickly removed and frozen at −20°C for 15 min. Subsequently, each frozen brain was rapidly cut into 5 coronal sections (2 mm thick). The sections were stained with 2% TTC (Servicebio Biotechnology, Ltd., Wuhan, China) for 20 min at 37°C in the dark and then fixed in 4% paraformaldehyde at 4°C for 24 h. Normal tissue stained red, and infarct tissue appeared white. The sections were photographed, and the photos were analysed using ImageJ 6.0 analysis software.

Percentage of infarct volume = total lesion volume/total brain volume × 100%.

### 2.5. Nissl's Staining

At 1, 3, and 7 d of reperfusion, 5 rats from each group were randomly chosen and anesthetized with 1% pentobarbital sodium (50 mg/kg, intraperitoneally). Brains were rapidly removed and fixed in 4% paraformaldehyde for 72 h and embedded in paraffin following standard methods. The paraffin-embedded blocks were cut into a series of 5 *μ*m thick slices that contained damaged cerebral cortex and striatum and stained with 1% toluidine blue (Servicebio Biotechnology, Ltd., Wuhan, China). The morphology of neurons and staining of Nissl bodies in the ischemic cortex were observed (×400 magnification) under a light microscope (DM4000B, Leica Microsystems Inc., Germany). Three random fields were chosen in each slice. An investigator blinded to the experimental design analysed the slides. The number of neurons stained positively by Nissl was counted to evaluate neuronal cell loss in the ischemic cortex.

### 2.6. TUNEL Staining

At 1, 3, and 7 d of reperfusion, 5 rats from each group were randomly chosen. The preparation of paraffin sections of brains was described above. A 50 *μ*l volume of TdT reaction mix (including 45 *μ*l Equilibration Buffer plus 5 *μ*l Nucleotide Mix and 1 *μ*l rTdT enzyme) (Promega Corporation, Wisconsin, USA) was added followed by incubation at 37°C for 1 h in the dark. Then, the slices were stained with DAPI at 37°C for 10 min; neutral balsam was added as the sealing agent in a dark room. The cerebral cortex was observed (×400 magnification) and photographed using a fluorescence microscope (DM5000B, Leica Microsystems Inc., Germany). Three random fields were chosen in each slice. An investigator blinded to the experimental design analysed the slides. The total number of cells and the number of TUNEL-positive cells were counted to evaluate the neuronal cell apoptosis rate.

Apoptosis rate = number of TUNEL-positive cells/total number of cells × 100%.

### 2.7. Western Blot Analysis

**Figure 2:**
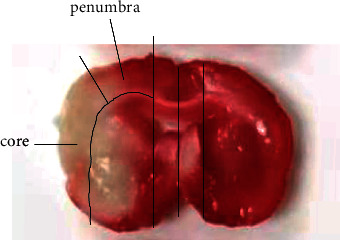
Isolation of the ischemic core and penumbra in the rat brain.

The core, penumbra, and contralateral areas of rat brains were rapidly isolated 3 d after reperfusion according to the method of Ashwal et al. [22]. The anterior and posterior brain tissues were coronally resected with a thickness of 3 mm from the frontal pole to the occipital pole, leaving the middle part. We made a longitudinal cut (from top to bottom) approximately 2 mm from the midline through each hemisphere and then made a transverse diagonal cut at approximately the “10 o'clock” position to separate the core from the penumbra (Figure 2).

Total protein was extracted from brain tissue with protein lysate (Beyotime Biotechnology, Ltd., Beijing, China). The concentration of protein was quantified using the BCA method. Protein samples (30 *μ*g) were separated on 12% SDS-polyacrylamide gels and transferred onto PVDF membranes (Millipore, Germany). Diluted primary antibodies against Beclin-1 (1 : 1000; CST, USA), LC3 (1 : 1000; Sigma, USA), and *β*-actin primary antibody (1 : 2000; Servicebio Biotechnology, Ltd., Wuhan, China) were added, and the sample was incubated overnight at 4°C. The membrane was rinsed thrice with TBST the next day and placed in a secondary antibody (1 : 3000; Servicebio Biotechnology, Ltd., Wuhan, China). The bands were visualized and photographed using a multimodal imaging platform (Fusion FX 5 Spectra, Vilber, France). Their intensities were determined using Vision Capt software. The expression of the target proteins was quantified and reported as the ratio of Beclin-1/*β*-actin and LC3 II/LC3 I intensities.

### 2.8. Transmission Electron Microscopy

Electron microscopy (EM) was performed on penumbras from rats subjected to 2 h of ischemia, followed by 3 d of reperfusion and BHD treatment (*n* = 3 per group). The rats were anesthetized; then, the brains were removed, and the penumbra cortex region was rapidly isolated, cut into small sections, and stored in 2.5% glutaraldehyde in 0.1 mol/L PBS (pH 7.4). The sections were postfixed with 1% osmium acid for 1 h, rinsed twice with PBS, dehydrated in graded ethanol, dried in acetone to a critical point, and then embedded in epoxy resin. Ultrathin sections (80 nm) were cut and viewed under a transmission electron microscope (HT7700, Hitachi, Ltd., Japan).

### 2.9. Statistical Analysis

Data were expressed as mean ± SD. Statistical analysis was performed using IBM SPSS Statistics 23 software. Two-way ANOVA was performed for the cerebral infarct volume, apoptosis rate, and number of neurons at different time points. The data were analysed by one-way ANOVA followed by LSD test when only three groups at one time point were analysed. Differences were considered statistically significant at *P* < 0.05.

## 3. Results

### 3.1. Quality Control of BHD

**Figure 3:**
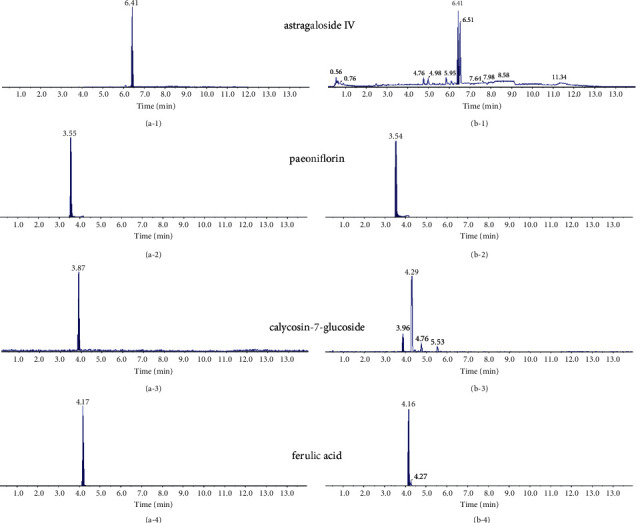
Quality control of BHD preparation by HPLC-MS analysis (a, b). (a) The result of the standards and (b) the result of the samples. Four representative bioactive ingredients were identified in BHD: a-1/b-1 astragaloside IV, a-2/b-2 paeoniflorin, a-3/b-3 calycosin-7-glucoside, and a-4/b-4 ferulic acid.

According to the Chinese Pharmacopoeia and previous reports [23], four representative bioactive ingredients were identified in BHD using HPLC-MS analysis (Figure 3). These ingredients included astragaloside IV, paeoniflorin, calycosin-7-glucoside, and ferulic acid, and their contents were 0.133, 0.492, 0.139, and 0.04 mg/g dry weight of raw materials, respectively.

### 3.2. The MCAO Model Was Successfully Established

**Figure 4:**
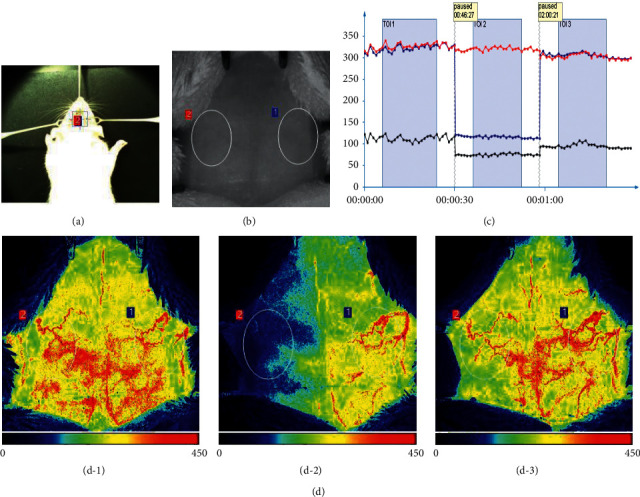
Standard of MCAO establishment. (a) Physical image of cerebral blood flow detection. (b) Black-and-white image corresponding to the physical image. (c) Line chart of the change in cerebral blood flow. (d) Cerebral blood flow perfusion map recorded by the PSI system before MCAO (d-1), 5 min after ischemia (d-2), and 5 min after reperfusion (d-3).

To be considered a successfully established MCAO model, the regional cerebral blood flow must be blocked by at least 65%, and the Zea Longa Neurological Score should be within 1–3 points. In this study, the blood flow on the ischemic side postischemic was reduced by approximately 66% compared with that preischemic, and the levels returned to greater than 90% after reperfusion (Figure 4). In addition, the neurofunctional score of rats with MCAO/R was 2.29 ± 0.66, and then the rats were randomly divided into an I/R group and an I/R + BHD group.

### 3.3. Effect of BHD on Cerebral Infarct Volume in Rats with Cerebral I/R Injury

**Figure 5:**
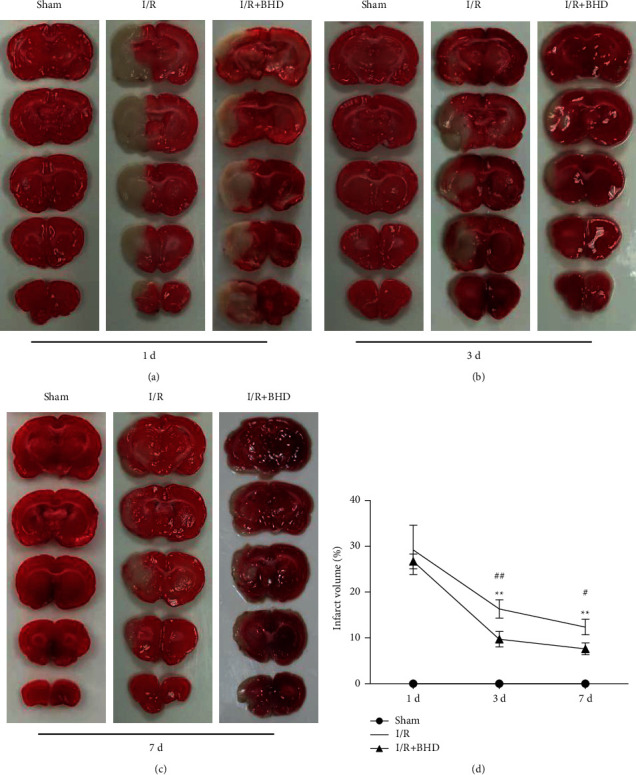
Infarction volume of rats in different groups at 1, 3, and 7 d after reperfusion, respectively. (a–c) Serial coronal brain sections by TTC staining in each group at 1 d (a), 3 d (b), and 7 d (c). (d) Statistical analysis of cerebral infarct volume in different groups (n = 3). ^∗^*P* < 0.05 and ^∗∗^*P* < 0.01 versus sham; ^#^*P* < 0.05 and ^##^*P* < 0.01 versus I/R.

The infarct volume was examined by TTC staining at each time point after reperfusion (Figure 5). The sham group showed uniform red staining, and no pale infarct area was noted at any time point. White infarct areas were observed in the I/R groups and I/R + BHD groups at 1, 3, and 7 d after reperfusion. At 1 d, no significant difference in infarct volume was noted between the I/R group and the I/R + BHD group (*P* > 0.05). However, the infarct volume in the I/R + BHD group was remarkably reduced compared with that in the I/R group after 3 d (*P* < 0.01) and 7 d (*P* < 0.05) of treatment. Furthermore, the differences in cerebral infarct volume between the I/R group and the I/R + BHD group at 3 and 7 d were 6.60 ± 3.05 and 4.76 ± 0.57, respectively. Thus, the protective effect of BHD on cerebral infarction is better after 3 d of treatment compared with 7 d.

### 3.4. Effect of BHD on Neuronal Apoptosis in Rats with Cerebral I/R Injury

**Figure 6:**
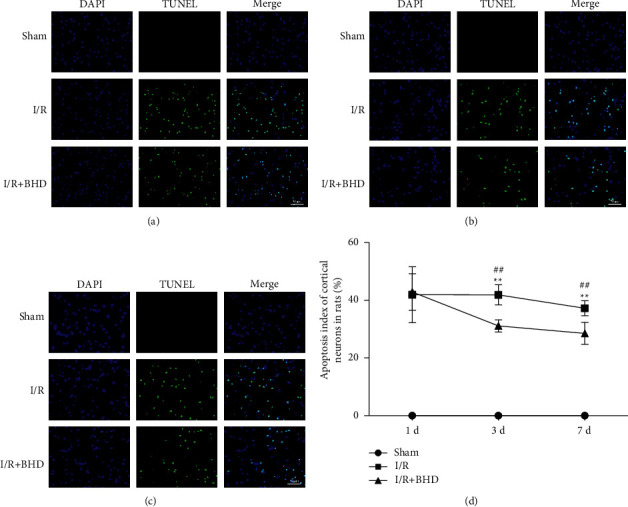
Neuronal apoptosis of rats in different groups at 1, 3, and 7 d postreperfusion. (a–c) TUNEL staining of ischemic lateral brain tissue of rats in each group at 1 d (a), 3 d (b), and 7 d (c) (scale bar = 50 *μ*m). (d) Statistical analysis of the apoptosis index of the cortices of rats in different groups (n = 5). ^∗^*P* < 0.05 and ^∗∗^*P* < 0.01 versus sham; ^#^*P* < 0.05 and ^##^*P* < 0.01 versus I/R.

TUNEL staining of cerebral slices (Figure 6) showed that the double-dyed fluorescent signal was minimally visible in the brains of the sham group at all time points. However, apoptosis appeared in the I/R groups and I/R + BHD groups at 1, 3, and 7 d of reperfusion. At 1 d of reperfusion, the difference between the I/R group and I/R + BHD group was not significant (*P* > 0.05). However, the apoptosis rate in brain cortex of the I/R + BHD group was dramatically reduced compared with that of the I/R group after 3 d (*P* < 0.01) and 7 d (*P* < 0.01) of reperfusion. Furthermore, the differences in the apoptosis rate between the I/R and I/R + BHD groups at 3 and 7 d were 10.73 ± 3.96 and 8.70 ± 2.21, respectively. Therefore, BHD is a more effective treatment on neuronal apoptosis when used for 3 d after reperfusion compared with 7 d.

### 3.5. BHD Improve Pathological Damage of Brain Tissue at Different Time Points of Cerebral I/R Injury

**Figure 7:**
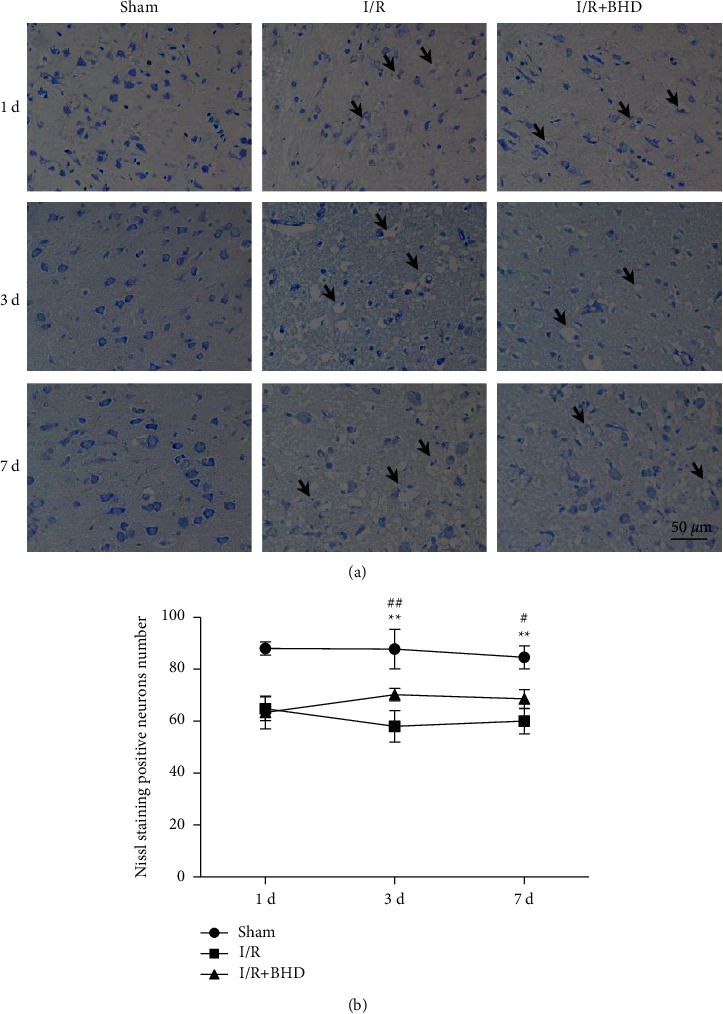
Morphological changes of neurons detected by Nissl staining. (a) Nissl staining of the ischemic cortex of rats in each group at 1, 3, and 7 d postreperfusion (scale bar = 50 *μ*m). (b) Statistical analysis of Nissl-positive neuron number in the different groups (n = 5). ^∗^*P* < 0.05 and ^∗∗^*P* < 0.01 versus sham; ^#^*P* < 0.05 and ^##^*P* < 0.01 versus I/R.

Figure 7 shows the results of Nissl staining. In the sham group at all time points, neurons in the brain tissue were arranged neatly and tightly, and the nuclei were clear and vacuole-like. The Nissl bodies were blue, dark stained and rich in number. In the I/R group, neurons on the ischemic side were seriously damaged, irregularly arranged, and atrophied. In addition, Nissl bodies became lightly stained, some neurons disappeared, and the intercellular space increased at 1, 3, and 7 d of I/R (*P* < 0.01). However, in the I/R + BHD group, the morphological changes of neurons were alleviated at 3 and 7 d, and the arrangement of neurons was more compact. In addition, the number of neurons stained positive in the I/R + BHD group was significantly increased compared with that in the I/R group after 3 d (*P* < 0.01) and 7 d (*P* < 0.05). Furthermore, the differences in neuron numbers between the I/R group and I/R + BHD group at 3 d and 7 d were 12.2 ± 3.70 and 8.6 ± 3.78, respectively. Thus, the protective effect of BHD on neuronal damage was better after 3 d compared with 7 d.

### 3.6. Effect of BHD on the Level of Autophagy-Related Proteins in Different Injured Areas after Cerebral I/R Injury

**Figure 8:**
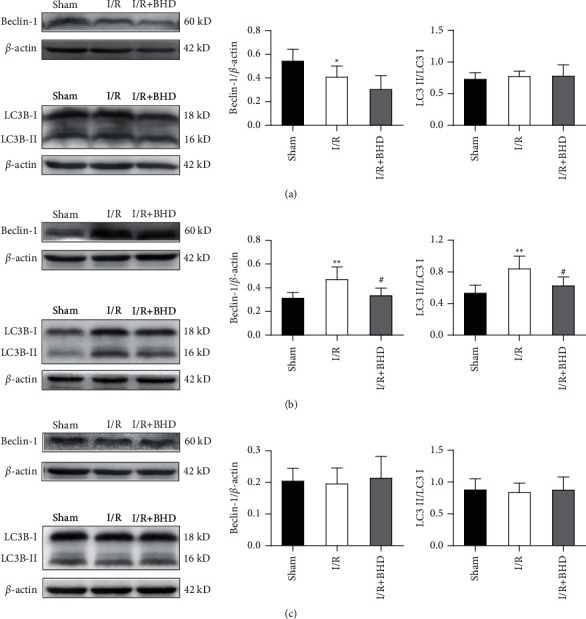
Western blotting analysis of Beclin-1 and LC3 levels at 3 d after reperfusion in different areas. a-c Statistical analysis of Beclin-1 and LC3 levels in the core (a), penumbra (b), and contralateral area (c) (*n* = 6). ^∗^*P* < 0.05 and ^∗∗^*P* < 0.01 versus sham; ^#^*P* < 0.05 and ^##^*P* < 0.01 versus I/R.

To explore whether autophagy was involved in the protective effect of BHD on cerebral I/R injury, we analysed Beclin-1 and LC3 levels in different areas 3 d after reperfusion, the time point for optimal protection (Figure 8). In the ischemic core, Beclin-1 expression was significantly diminished in the I/R group compared with the sham group (*P* < 0.05), and no significant change was noted between the I/R group and the I/R + BHD group. In addition, no dramatic difference in transformation of LC3 I to LC3 II was noted among the three groups. In the penumbra, cerebral I/R injury markedly increased the Beclin-1 levels (*P* < 0.01), significantly promoted the degradation of LC3 (*P* < 0.01), and activated autophagy. Nevertheless, in the I/R + BHD group, BHD significantly prevented Beclin-1 expression (*P* < 0.05) and decreased the accumulated amount of LC3 II compared with the I/R group (*P* < 0.05). In the contralateral area, the level of Beclin-1 and the transformation of LC3 I to LC3 II were not significantly different among the three groups.

### 3.7. Observation of Damage and Autophagy of Neurons in Penumbra by TEM

**Figure 9:**
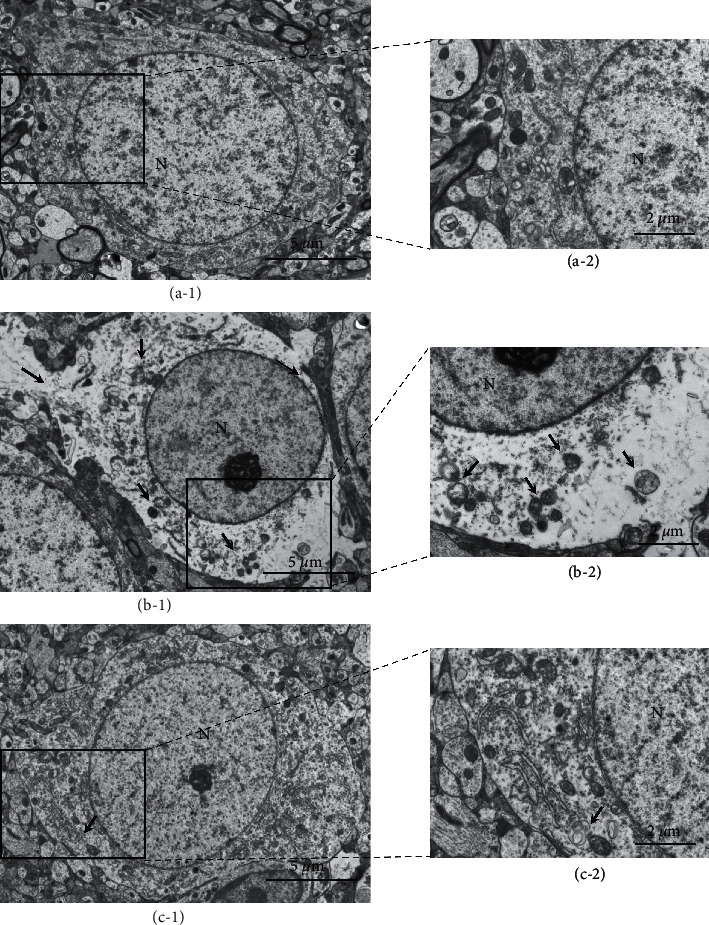
Ultrastructure of neurons in the penumbra of rats in each group observed by TEM at 3 d after reperfusion. (a–c) Neuronal damage and autophagy observed by TEM in the sham group (a-1) (a-2), I/R group (b-1) (b-2), and I/R + BHD group (c-1) (c-2). Scale bar = 5 *μ*m in (a-1), (b-1), and (c-1); scale bar = 2 *μ*m in (a-2), (b-2), and (c-2).

To verify the autophagy regulating effect of BHD in the penumbra of rats at 3 d after MCAO/R, we observed neurons in the penumbra of the cerebral cortex by TEM (Figure 9). In the sham group, there was abundant intracellular matrix, and the nuclear membrane was intact. No typical autophagy was found in the neurons. In the I/R group, the neurons showed severe oedema and local damage to the cell membrane. Lipofuscin and a certain amount autophagosome were observed. In the I/R + BHD group, the neurons showed mild oedema and intact cell membranes. Individual autophagic lysosomes were noted. The degree of autophagy in the I/R + BHD group was lower than that in the I/R group.

## 4. Discussion

BHD is a classical TCM prescription and is widely used in the clinical treatment of ischemic stroke in China. Moreover, BHD has also been shown to have neuroprotective effects in an animal model of MCAO/R [23]. In our study, we evaluated the neuroprotective effects of BHD at 1, 3, and 7 d after reperfusion. The data demonstrated that BHD can significantly reduce cerebral infarct volume, apoptosis rate, and neuron damage at 3 d and 7 d after reperfusion. However, we observed that rats in the model group showed some degree of self-recovery at 7 d, and the protective effect of BHD on cerebral I/R injury was better at 3 d compared with 7 d. Therefore, 3 d were chosen as the time point to explore the specific mechanism by which BHD inhibits cerebral I/R injury.

An increasing number of studies have shown that autophagy plays an important role during cerebral I/R injury and is frequently mediated by oxidative stress, endoplasmic reticulum stress, and other reactions in cells after MCAO/R [24, 25]. BHD plays a protective role against ischemic stroke with multiple targets, such as oxidative stress, endoplasmic reticulum stress, inflammation, and apoptosis. We thus hypothesized that autophagy might be one of the important mechanisms of the neuroprotective effect of BHD on cerebral I/R injury. We first examined the expression of the autophagy-related proteins Beclin-1 and LC3 in the core, penumbra, and contralateral areas 3 d after MCAO/R and BHD administration. The results showed that in the penumbra, the autophagy-associated proteins Beclin-1 and LC3 II/I were upregulated after cerebral I/R injury. These results are consistent with previous studies. A study demonstrated that LC3 II/I and Beclin-1 levels in the penumbra of rats in the I/R group were significantly increased compared with those in the sham group at 3 d after reperfusion after ischemia for 2 h [26]. Moreover, another study also showed that Beclin-1 protein and LC3B protein levels markedly increased in model groups with 2 h ischemia followed by 72 h reperfusion compared with the sham group. In addition, 1–3 d after operation, the expression remained at a high level [27]. However, one study reported results that are inconsistent with the trend noted above. At 24 h and 7 d after MCAO/R with 1.5 h ischemia, Beclin-1 and LC3 II levels significantly decreased in the ischemic penumbra [28]. In addition, our study found that in the core, Beclin-1 was downregulated in the I/R group compared with the sham group, and LC3 II/I was not significantly different between the sham and I/R groups. However, these results differ from those of a previous study in which Beclin-1 and LC3-II expression were significantly decreased in the ischemic core, as detected at 24 h and 7 d after reperfusion after MCAO for 1.5 h [28]. These discrepancies may result from different times of ischemia and reperfusion in the model. In addition, in the contralateral area, the degree of autophagy was not significantly different between the sham and I/R groups. After treatment with BHD, Beclin-1 levels and the transformation of LC3 I to LC3 II were only significantly lower in the penumbra compared with that noted in the I/R group. However, no significant difference in autophagy degree was noted between the I/R group and I/R + BHD group in the core and contralateral areas. This result indicated that BHD exerted a neuroprotective effect on rats with cerebral I/R injury by inhibiting neuronal autophagy in the penumbra of rats 3 d after MCAO/R. The results of autophagy in neurons of the penumbra cortex observed by TEM confirmed the viewpoint noted above.

Collectively, our study suggested that autophagy was involved in the neuroprotective effect of BHD on cerebral I/R injury, which primarily occurred in the penumbra. However, autophagy inhibitors or activators were not used in this study, and these agents could be used to more accurately explain the role of autophagy in cerebral I/R injury. Thus, in further research, we will examine the effect of BHD on autophagy during different time in the process of cerebral I/R injury using autophagy inhibitors or activators. Furthermore, autophagy is a dynamic and complex process, and autophagy and apoptosis are events with close interactions [29]. Therefore, the exact regulation of every part of the autophagy process by BHD and the interaction between autophagy and apoptosis is worthy of further study.

## 5. Conclusions

In summary, our study found that continuous administration of BHD for 3 d and 7 d after reperfusion can alleviate the brain injury caused by cerebral I/R in rats. Furthermore, we chose 3 d as the experimental time point to explore the neuroprotective mechanism of BHD on cerebral I/R injury and found that BHD primarily suppresses autophagy in the penumbra area. The results provide theoretical support for the clinical application of BHD.

## Data Availability

The data used to support the findings of this study are available from the corresponding author upon request.

## References

[1] Sun Y., Zhu Y., Zhong X., Chen X., Wang J., Ying G. (2018). Crosstalk between autophagy and cerebral ischemia. *Frontiers in Neuroscience*.

[2] Fricker M., Tolkovsky A. M., Borutaite V., Coleman M., Brown G. C. (2018). Neuronal cell death. *Physiological Reviews*.

[3] Wu M. Y., Yiang G. T., Liao W. T. (2018). Current mechanistic concepts in ischemia and reperfusion injury. *Cellular Physiology and Biochemistry*.

[4] Amani H., Mostafavi E., Alebouyeh M. R. (2019). Would colloidal gold nanocarriers present an effective diagnosis or treatment for ischemic stroke?. *International Journal of Nanomedicine*.

[5] Xu D., Kong T., Zhang S., Cheng B., Chen J., Wang C. (2021). Orexin-a protects against cerebral ischemia-reperfusion injury by inhibiting excessive autophagy through OX1R-mediated MAPK/ERK/mTOR pathway. *Cellular Signalling*.

[6] Zhu J., Wang L., Zhang J. (2020). Galuteolin Inhibited autophagy for neuroprotection against transient focal cerebral ischemia in rats. *NeuroMolecular Medicine*.

[7] Wang P., Shao B. Z., Deng Z., Chen S., Yue Z., Miao C. Y. (2018). Autophagy in ischemic stroke. *Progress in Neurobiology*.

[8] Gabryel B., Kost A., Kasprowska D. (2012). Neuronal autophagy in cerebral ischemia--a potential target for neuroprotective strategies. *Pharmacological Reports*.

[9] Zhang D. M., Zhang T., Wang M. M. (2019). TIGAR alleviates ischemia/reperfusion-induced autophagy and ischemic brain injury. *Free Radical Biology and Medicine*.

[10] Shao Z. Q., Dou S. S., Zhu J. G. (2021). Apelin-13 inhibits apoptosis and excessive autophagy in cerebral ischemia/reperfusion injury. *Neural Regeneration Research*.

[11] Li F., Yang B., Li T., Gong X., Zhou F., Hu Z. (2019). HSPB8 over-expression prevents disruption of blood-brain barrier by promoting autophagic flux after cerebral ischemia/reperfusion injury. *Journal of Neurochemistry*.

[12] Peng C., Rao W., Zhang L. (2018). Mitofusin 2 exerts a protective role in ischemia reperfusion injury through increasing autophagy. *Cellular Physiology and Biochemistry*.

[13] Sun B., Ou H., Ren F. (2018). Propofol inhibited autophagy through Ca(2+)/CaMKKbeta/AMPK/mTOR pathway in OGD/R-induced neuron injury. *Molecular Medicine*.

[14] Zheng Y., Wu Z., Yi F. (2018). By activating Akt/eNOS bilobalide B inhibits autophagy and promotes angiogenesis following focal cerebral ischemia reperfusion. *Cellular Physiology and Biochemistry*.

[15] Huang H., Mu Q., Liu P., Hu X., Gao H., Zheng X. (2014). Neuroprotective effects of buyang huanwu decoction on cerebral ischemia-induced neuronal damage. *Neural Regeneration Research*.

[16] She Y., Shao L., Zhang Y. (2019). Neuroprotective effect of glycosides in buyang huanwu decoction on pyroptosis following cerebral ischemia-reperfusion injury in rats. *Journal of Ethnopharmacology*.

[17] Sun H., Wu C. (2019). Acupuncture combined with buyang huanwu decoction in treatment of patients with ischemic stroke. *Journal of International Medical Research*.

[18] Jin Y. L., Dong L. Y., Wu C. Q. (2013). Buyang huanwu decoction fraction protects against cerebral ischemiareperfusion injury by attenuating the inflammatory response and cellular apoptosis. *Neural Regeneration Research*.

[19] Kong X., Su X., Zhu J. (2014). Neuroprotective effect of buyang huanwu decoction on rat ischemic/reperfusion brain damage by promoting migration of neural precursor cells. *Rejuvenation Research*.

[20] Yang J., Gao F., Zhang Y., Liu Y., Zhang D. (2015). Buyang huanwu decoction (BYHWD) enhances angiogenic effect of mesenchymal stem cell by upregulating vegf expression after focal cerebral ischemia. *Journal of Molecular Neuroscience*.

[21] Zheng X. W., Shan C. S., Xu Q. Q. (2018). Buyang huanwu decoction targets SIRT1/VEGF pathway to promote angiogenesis after cerebral ischemia/reperfusion injury. *Frontiers in Neuroscience*.

[22] Ashwal S., Tone B., Tian H. R., Cole D. J., Pearce W. J. (1998). Core and penumbral nitric oxide synthase activity during cerebral ischemia and reperfusion. *Stroke*.

[23] Dou B., Zhou W., Li S. (2018). Buyang huanwu decoction attenuates infiltration of natural killer cells and protects against ischemic brain injury. *Cellular Physiology and Biochemistry*.

[24] Sun X., Liu H., Sun Z. (2020). Acupuncture protects against cerebral ischemia-reperfusion injury via suppressing endoplasmic reticulum stress-mediated autophagy and apoptosis. *Molecular Medicine*.

[25] Yang B., Zang L. E., Cui J. W., Zhang M. Y., Ma X., Wei L. -L. (2020). Melatonin plays a protective role by regulating miR-26a-5p-NRSF and JAK2-STAT3 pathway to improve autophagy, inflammation and oxidative stress of cerebral ischemia-reperfusion injury. *Drug Design, Development and Therapy*.

[26] Pan G., Jin L., Shen W. (2020). Treadmill exercise improves neurological function by inhibiting autophagy and the binding of HMGB1 to Beclin1 in MCAO juvenile rats. *Life Science*.

[27] Shu S., Li C. M., You Y. L., Qian X. L., Zhou S., Ling C. Q. (2016). Electroacupuncture ameliorates cerebral ischemia-reperfusion injury by regulation of autophagy and apoptosis. *Evid Based Complement Alternative Medicine*.

[28] Wu M., Zhang H., Kai J. (2018). Rapamycin prevents cerebral stroke by modulating apoptosis and autophagy in penumbra in rats. *Annals of Clinical and Translational Neurology*.

[29] Maiuri M. C., Criollo A., Kroemer G. (2010). Crosstalk between apoptosis and autophagy within the beclin 1 interactome. *The EMBO Journal*.

